# Identification of epiretinal proliferation in various retinal diseases and vitreoretinal interface disorders

**DOI:** 10.1186/s40942-020-00233-0

**Published:** 2020-07-10

**Authors:** Ismael Chehaibou, Moritz Pettenkofer, Andrea Govetto, Gilad Rabina, SriniVas R. Sadda, Jean-Pierre Hubschman

**Affiliations:** 1 Retina Division, Stein Eye Institute, University of California Los Angeles, 100 Stein Plaza, Los Angeles, CA 90095 USA; 2 Ophthalmology Department, AP-HP, Université de Paris, Hôpital Lariboisière, 75010 Paris, France; 3 Ophthalmology Department, Fatebenefratelli-Oftalmico Hospital, ASST-Fatebenefratelli-Sacco, Milan, Italy; 4 Department of Ophthalmology, Tel Aviv Sourasky Medical Center, Affiliated to the Sackler Faculty of Medicine, Tel Aviv University, Tel Aviv, Israel; 5 Doheny Image Reading Center, Doheny Eye Institute, Los Angeles, CA USA

**Keywords:** Epiretinal membrane, Epiretinal proliferation, Full-thickness macular holes, Lamellar hole-associated epiretinal proliferation, Lamellar macular holes, Macular edema, Müller glial cells, Spectral-domain optical coherence tomography

## Abstract

**Background:**

To describe the presence of epiretinal proliferation in eyes with various retinal and vitreoretinal interface conditions.

**Methods:**

Consecutive patients seen at the Stein Eye Institute, by one retina specialist, from December 2018 to March 2019, and demonstrating epiretinal proliferation on optical coherence tomography (OCT) were enrolled in this cross-sectional study. Included patients were divided into two groups: vitreoretinal interface pathologies group or retinal diseases group. Presence of epiretinal proliferation and its localization within the 9 macular sectors, as defined by the Early Treatment Diabetic Retinopathy Study (ETDRS), were assessed on OCT.

**Results:**

77 eyes from 69 patients demonstrated epiretinal proliferation on OCT. The most frequently involved ETDRS sector was the 1-mm central subfield, followed by inner temporal and inner nasal sectors. Localization of epiretinal proliferation correlated with the presence of any retinal abnormalities in the same quadrant (r = 0.962; P < 0.0001). 31 eyes (40.3%) demonstrated symptomatic vitreoretinal interface pathologies including lamellar macular hole, full-thickness macular hole, epiretinal membrane and history of macular peeling. 46 eyes (59.7%) manifested various retinal diseases, including age-related macular degeneration, diabetic retinopathy, refractory macular edema, vein occlusion and high myopia.

**Conclusions:**

Epiretinal proliferation was noted in several retinal conditions and not limited only to full-thickness and lamellar macular holes. Different mechanisms affecting retinal homeostasis might trigger Müller cells dysregulation, potentially leading to abnormal retinal remodeling.

## Introduction

Epiretinal proliferation (ERP) has recently been described in eyes with lamellar macular holes [1]. This proliferation was distinguished from epiretinal membrane (ERM) on spectral-domain optical coherence tomography (SD-OCT), and defined as an isoreflective space-filling material over the retinal surface, often delimited by a thin highly reflective line, and without tractional properties [1, 2]. Thereafter, this proliferative tissue has also been identified in eyes with full-thickness macular holes (FTMH) and in association with ERM development [3, 4].

While initially suspected to originate from the vitreous, Pang and co-workers noticed that ERP contains mainly Müller glial cells, which makes its genesis from retinal tissue very likely [2, 5]. However, the initial trigger leading to this proliferative process remains poorly understood. Since Müller cells may play a central role in retinal homeostasis, we hypothesized that other macular pathologies, besides LMH and FTMH, might also develop such proliferative tissue. Thus, the purpose of this study was to identify the presence of ERP in association with various retinal disorders, and to describe its morphological characteristics on SD-OCT.

## Methods

### Design

This cross-sectional and descriptive study adhered to the principles of the Declaration of Helsinki and to the regulations of the Health Insurance Portability and Accountability Act. This study was approved by Institutional Review Board of the University of California Los Angeles Office of Human Research Protection (IRB#16-000574). All consecutive patients examined by a single retina specialist (JPH), at the Stein Eye Institute of the University of California Los Angeles, from December 2018 to March 2019, and demonstrating ERP on SD-OCT were enrolled in this study. The main exclusion criteria were uninterpretable or poor-quality SD-OCT scans. Epiretinal proliferation was defined on SD-OCT as a homogenous, non-contractile, iso-reflective layer, and often surrounded by a thin hyperreflective band, as previously described in LMH [1].

### Patients

Patient’s clinical charts were reviewed to record significant ocular history, and clinical examination findings including visual acuity (VA) and lens status. Included eyes were divided into two groups: vitreoretinal interface (VRI) pathologies group, which included LMH, FTMH, idiopathic ERM and iatrogenic retinal macular defect, and retinal diseases group which included age-related macular degeneration (AMD), diabetic retinopathy, retinal vein occlusion, refractory chronic macular edema and high myopia.

### Spectral-domain OCT

**Fig. 1:**
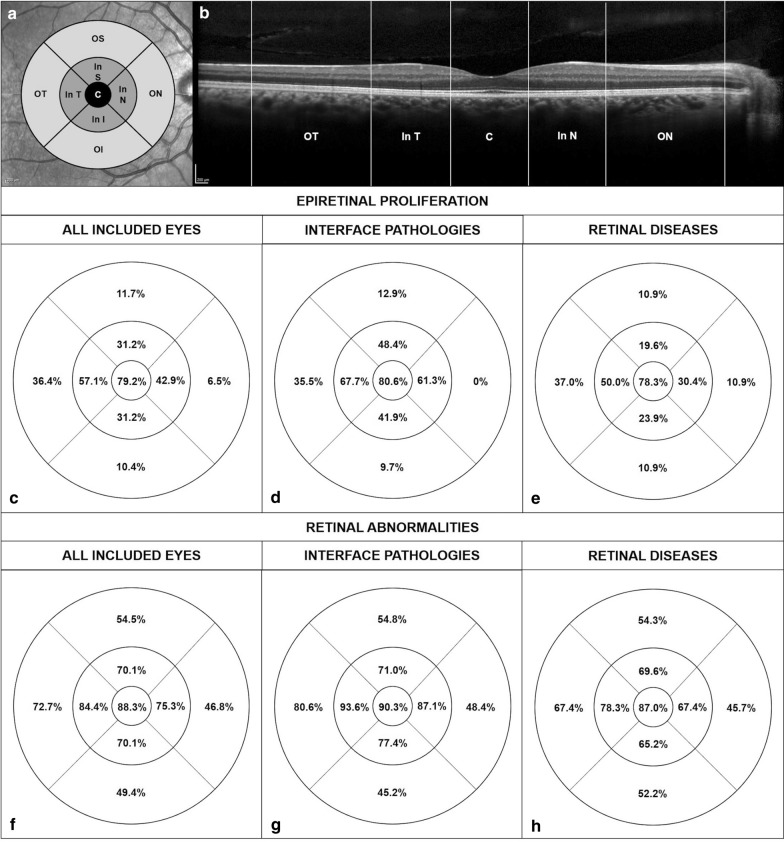
Localization of epiretinal proliferation and retinal abnormalities. **a** The standard ETDRS quadrants dividing the macula into central fovea, inner macula, and outer macula. C: Central foveal quadrant, In S: Inner superior macula, In N: Inner nasal macula, In I: Inner inferior macula, In T: Inner temporal macula, OS: Outer superior macula, ON: Outer nasal macula, OI: Outer inferior macula, OT: Outer temporal macula. **b** Presence of epiretinal proliferation (ERP) was localized on optical coherence tomography (OCT) scans using the 9 ETDRS quadrants. The second row shows the rate of eyes displaying ERP within the 9 ETDRS sectors in all included eyes (**c**), in eyes with vitreoretinal interface (VRI) pathologies (**d**) and in eyes treated for different retinal diseases (**e**). The most frequently involved ETDRS sector was the 1-mm central circle, followed by the inner temporal and inner nasal quadrants. The third row shows the rate of eyes with retinal abnormalities including partial or full-thickness retinal defect, epiretinal membrane, intraretinal cysts, pigmentary epithelium detachment, and outer retinal layers disruption, within the nine ETDRS sectors, among all included eyes (**f**), in eyes with vitreoretinal interface pathologies (**g**) and in eyes treated for different retinal diseases (**h**). Localization of ERP within the 9 ETDRS sectors was strongly correlated with the presence in the same sector of any retinal lesion in all the studied eyes (r = 0.962; P < 0.0001), in the VRI pathologies group (r = 0.938; P = 0.0002) and in the retinal diseases group (r = 0.909; P = 0.001)

All included eyes were imaged with the Spectralis SD-OCT device (Heidelberg Engineering GmbH, Heidelberg, Germany), and reviewed with the Heidelberg Eye Explorer (version 1.8.6.0) using the HRA/Spectralis Viewing Module (version 5.8.3.0). All OCT scans were analyzed by two graders (MP, IC) to assess the presence of ERP and its localization within the 9 macular sectors, as defined by the Early Treatment Diabetic Retinopathy Study (ETDRS). The foveal central subfield corresponding to the inner 1-mm-diameter circle was considered as an individual quadrant; the inner circle subfield was located between the inner and middle 3-mm-diameter circles; and the outer circle subfield between the middle and outer 6-mm-diameter circles (Fig. 1). Both the inner and the outer circles subfield were subdivided into superior, nasal, inferior, and temporal quadrants. Presence of a partial or full-thickness retinal defect, epiretinal membrane, macular edema, pigmentary epithelium detachment (PED), laser scars and outer retinal layers (external limiting membrane and/or ellipsoidal zone) disruption were recorded, and their topographic distribution was correlated with the presence of ERP within the 9 ETDRS sectors. Epiretinal membranes were graded using the staging scheme proposed by Govetto and colleagues [6].

### Statistical analysis

Descriptive statistics were obtained using XLSTAT software version 2018.1.49572 (Assinsoft, Paris, France). Quantitative values are presented as mean ± standard deviation (SD), while qualitative values are listed as ratio and percentage. As this study was an investigative observational analysis, no comparative analyses were performed. Correlations between the location of ERP within the 9-ETDRS quadrants and the presence of SD-OCT retinal abnormalities were assessed using the Pearson’s correlation test. A P value of less than 0.05 was considered statistically significant.

## Results

Seventy-seven eyes of 69 patients, 39 (56.5%) female and 30 (43.5%) male, demonstrated ERP on SD-OCT examination and were enrolled in this study. Mean age of patients was 75.7 ± 13.1 years. Thirty-one (40.3%) eyes were included within the VRI group, while 46 (59.7%) eyes were included within the retinal diseases group.

Overall, ERP was noted in 3.1 ± 2.1 (range: 1–9) ETDRS sectors [2.4 ± 1.6 (range: 0–5) within the inner 1-mm-diameter circle or inner ETDRS circle subfield; and 0.6 ± 1.0 (range: 0–4) within the outer ETDRS circle subfield]. The most frequently involved retinal quadrant was the 1-mm central subfield (61/77 eyes, 79.2%), followed by the inner temporal quadrant (44/77 eyes, 57.1%) and the inner nasal quadrant (33/77 eyes, 42.9%). Localization of ERP within the 9 ETDRS sectors was strongly correlated with the presence in the same sector of any retinal lesion, as previously defined (r = 0.962; P < 0.0001) (Fig. 1).

### Vitreoretinal interface pathologies group

In the VRI pathologies group which includes 31 eyes, 12/31 (38.7%) were diagnosed with LMH, 4/31 (12.9%) with FTMH, 8/31 (25.8%) had idiopathic ERM, and 7/31 (22.6%) eyes had undergone prior macular peeling surgery. Clinical data and SD-OCT features of included eyes within the VRI group are reported in Table 1.

**Table 1:** Characteristics of the Eyes within the vitreoretinal interface pathologies group

	Lamellar macular hole	Full-thickness macular hole	Epiretinal membrane	Post-macular peeling	All eyes
N, eyes	12 (38.7%)	4 (12.9%)	8 (25.8%)	7 (22.6%)	31 (100.0%)
Clinical data					
Visual acuity, LogMAR (Snellen equivalent)	0.50 ± 0.54 (20/63)	0.52 ± 0.30 (20/66)	0.47 ± 0.63 (20/59)	0.24 ± 0.19 (20/34)	0.43 ± 0.48 (20/53)
Phakic lens status	3 (25.0%)	0 (0%)	1 (12.5%)	0 (0%)	4 (12.9%)
Glaucoma	3 (25.0%)	1 (25.0%)	3 (37.5%)	3 (42.9%)	10 (32.3%)
Retinal tear	0 (0%)	0 (0%)	0 (0%)	3 (42.9%)	3 (9.7%)
Retinal detachment	1 (8.3%)	1 (25.0%)	2 (25.0%)	1 (14.3%)	5 (16.1%)
SD-OCT features					
Macular PVD^a^	8/10 (80.0%)	1/1 (100.0%)	6/6 (100%)	–	15/17 (88.2%)
ERM	8 (66.7%)	1 (25.0%)	8 (100.0%)	3 (42.9%)	20 (64.5%)
ELM disruption^b^	8 (66.7%)	–	2 (25.0%)	4 (57.1%)	14/27 (51.9%)
EZ disruption^b^	9 (75.0%)	–	2 (25.0%)	4 (57.1%)	15/27 (55.6%)
N, ETDRS quadrants	4.6 ± 1.7	4.0 ± 2.7	2.6 ± 2.0	2.7 ± 2.4	3.6 ± 2.2

*N* number, *SD-OCT* spectral-domain optical coherence tomography, *PVD* posterior vitreous detachment, *ERM* epiretinal membrane, *ELM* external limiting membrane, *EZ* ellipsoidal zone, *ETDRS* early treatment diabetic retinopathy study

^a^Vitrectomized eyes are not included

^b^Full-thickness macular holes are not included

#### Lamellar macular hole and full-thickness macular hole

**Fig. 2:**
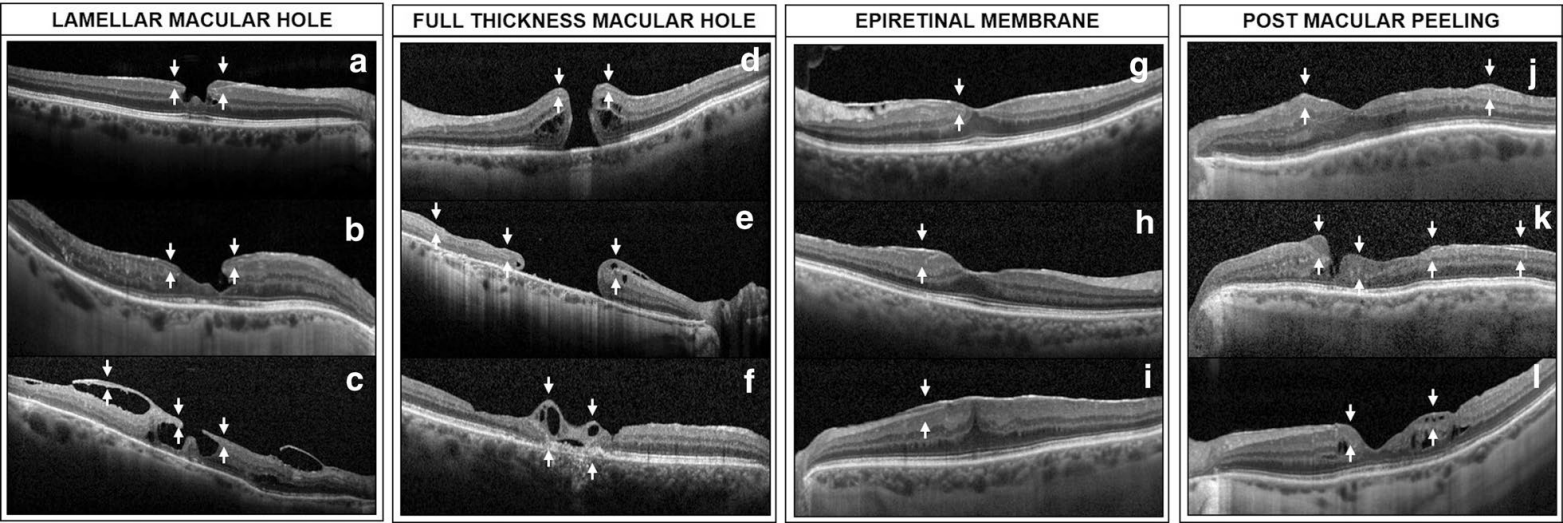
SD-OCT scans of eyes with vitreoretinal interface pathologies and epiretinal proliferation. **a**, **b** Lamellar macular holes (LMH) with epiretinal proliferation (ERP) (white arrows) over the edges of the foveal defect. This proliferative material was noted to be connected with the retinal layers within the hole. **c** LMH in the setting of advanced AMD, with attached posterior hyaloid in the macular area. Epiretinal proliferation was noted over the edges of the hole and spread out along the posterior hyaloid. **d** Patient with a stage 4 full-thickness macular hole (FTMH), and displaying ERP over the edges of the hole. **e** Optical coherence tomography (OCT) scans showing a chronic FTMH with ERP over the hole edges and over the temporal retinal surface. Intraretinal cysts were noted within the nasal edge of the macular hole, and within the ERP. **f** Patient with macular telangiectasia type 2 with a history of macular surgery for FTMH. Epiretinal proliferation with cystic cavities was noted over the macular hole. **g**–**i** OCT scans showing ERP in eyes with epiretinal membrane. The proliferative material was noted to be either above (**g**, **h**) or under (**i**) the ERM. **j** OCT scan showing ERP over the nasal and temporal macular area after ERM peeling. **k** Iatrogenic parafoveal inner retinal defect. Epiretinal proliferation was noted at the edges of the retinal defect, but also over the temporal retinal surface. **l** OCT scan after macular peeling for LMH. A proliferative material was observed over the nasal and temporal edges of the fovea. Cystic cavities involved both the retina and ERP

In all eyes with either LMH or FTMH, ERP was noted at the edges of the hole (Fig. 2a–f). In LMH eyes, this proliferative tissue appeared connected to the intraretinal layers, within the hole, in all of the 12 cases (Fig. 2b). A macular posterior vitreous detachment was noted in 8 out of 12 (66.7%) LMH eyes, while 2 (16.7%) had a vitreomacular adhesion and 2 (16.7%) had a history of vitrectomy for retinal detachment. Interestingly, in one LMH with vitreomacular adhesion, ERP appeared to be in continuity with the bottom of the hole and to spread out above the posterior hyaloid (Fig. 2c).

Two eyes manifested a stage 4 FTMH (Fig. 2d) and one eye had a chronic macular hole (Fig. 2e). The latter displayed intraretinal edema at the nasal edge of the retina also involving the overlying ERP. Finally, one FTMH eye was diagnosed with macular telangiectasia type 2 (MacTel), and had a history of FTMH surgery (Fig. 2f).

#### Idiopathic epiretinal membrane

Epiretinal proliferation was noted in association with idiopathic ERM in 8 eyes (3 stage 1, 3 stage 2, 2 stage 3 and 1 stage 4) (Fig. 2g–i). Based on SD-OCT analysis, the proliferative tissue was considered to be above the ERM in 6 out of 8 eyes, and between the retinal surface and the ERM in 2 out of 8 eyes.

#### Previous macular peeling surgery

Seven eyes displayed ERP after macular peeling surgery for ERM (4/7 eyes) (Fig. 2j), FTMH (2/7 eyes) and LMH (1/7 eyes). This proliferative material was noted in the parafoveal area, and collocated with a deep inner retinal defect (Fig. 2k) in 3 cases, and with dissociation of optic nerve fiber layer (DONFL) but without visible retinal defect in 2 cases. Finally, 2 eyes had irregular foveal contour after LMH (1 eye) and FTMH (1 eye) (Fig. 2l) surgery and demonstrated ERP in the 1-mm central area, which seemed to fill the irregularities of the retinal surface.

### Retinal conditions group

In the retinal diseases group, encountered pathologies were AMD (14/46 eyes, 30.4%), diabetic retinopathy (10/46 eyes, 21.2%), refractory chronic macular edema (10/46 eyes, 21.2%), vein occlusion (8/46 eyes, 17.4%) and high myopia (4 eyes, 8.7%). Clinical data and SD-OCT features of included eyes within the retinal diseases group are reported in Table 2.

**Table 2:** Characteristics of the eyes within the retinal conditions group

	AMD	Diabetic retinopathy	Refractory chronic CME	Vein occlusion	High myopia	All eyes
N, eyes	14 (30.4%)	10 (21.7%)	10 (21.7%)	8 (17.4%)	4 (8.7%)	46 (100.0%)
Clinical data						
Visual acuity, LogMAR	0.31 ± 0.22	0.47 ± 0.66	0.74 ± 0.69	0.30 ± 0.13	0.27 ± 0.20	0.43 ± 0.48
Phakic lens status	0 (0%)	2 (20.0%)	1 (10.0%)	0 (0%)	0 (0%)	3 (6.5%)
Glaucoma	3 (21.4%)	8 (80.0%)	4 (40.0%)	5 (62.5%)	1 (25.0%)	21 (45.7%)
History of macular edema	8 (57.1%)	10 (100.0%)	10 (100.0%)	8 (100.0%)	0 (0%)	36 (78.3%)
Intravitreal injections						
Anti-VEGF	7 (87.5%)	9 (90.0%)	5 (50.0%)	6 (75.0%)	0 (0%)	27 (58.7%)
Steroids	0 (0%)	2 (20.0%)	5 (50.0%)	2 (25.0%)	0 (0%)	9 (16.1%)
Laser treatment						
PRP	0 (0%)	9 (90.0%)	0 (0%)	6 (75.0%)	0 (0%)	15 (32.6%)
FML	0 (0%)	3 (33.3%)	0 (0%)	0 (0%)	0 (0%)	3 (6.5%)
Retinal tear	0 (0%)	1 (10.0%)	0 (0%)	0 (0%)	2 (50.0%)	3 (6.5%)
Retinal detachment	0 (0%)	0 (0.0%)	5 (50.0%)	0 (0%)	1 (25.0%)	6 (13.0%)
SD-OCT features						
Macular PVD^†^	10/12 (83.3%)	2/5 (40.0%)	3/3 (100.0%)	6/7 (85.7%)	3/4 (75.0%)	24/31 (77.4%)
ERM	7 (50.0%)	6 (60.0%	8 (80.0%)	5 (62.5%)	1 (25.0%)	27 (58.7%)
Macular edema	3 (21.4%)	4 (40.0%)	8 (80.0%)	2 (25.0%)	0 (0%)	17 (37.0%)
ELM disruption	10 (71.4%)	6 (60.0%)	6 (60.0%)	4 (50.0%)	3 (75.0%)	29 (63.0%)
EZ disruption	10 (71.4%)	9 (90.0%)	8 (80.0%)	5 (62.5%)	3 (75.0%)	35 (76.1%)
N, ETDRS quadrants	1.4 ± 0.6	4.0 ± 2.6	3.3 ± 2.5	3.6 ± 1.7	1.3 ± 0.5	2.7 ± 2.1

*N* number, *VEGF* vascular endothelial growth factor, *PRP* pan-retinal photocoagulation, *FML* focal macular laser, *SD-OCT* spectral domain optical coherence tomography, *PVD* posterior vitreous detachment, *ERM* epiretinal membrane, *ELM* external limiting membrane, *EZ* ellipsoidal zone, *ETDRS* early treatment diabetic retinopathy study

^†^Vitrectomized eyes are not included

**Fig. 3:**
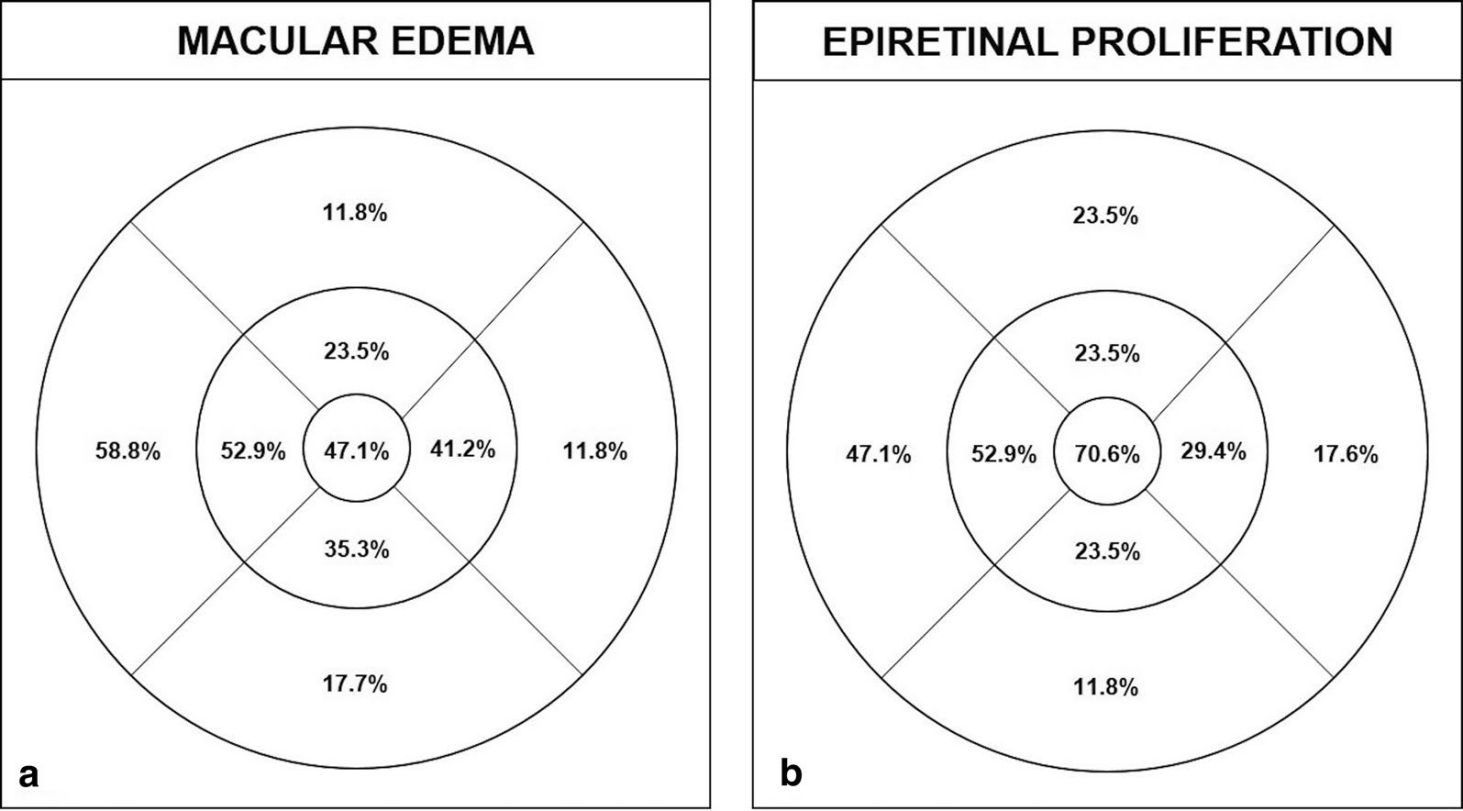
Localization of intraretinal cysts and epiretinal proliferation in eyes with macular edema. Seventeen eyes presented macular edema on spectral-domain optical coherence tomography (SD-OCT). Intraretinal cysts were noted to be present mainly in the 1-mm central ETDRS circle, and in the temporal inner and outer sectors (**a**). Distribution of epiretinal proliferation within the ETDRS sectors was noted to match with the presence of retinal cysts, with a strong correlation (r = 0.790; P = 0.011) (**b**)

Seventeen out of 46 (37.0%) eyes had macular edema on SD-OCT examination. There was a strong correlation between the localization of macular cysts and epiretinal proliferation within the 9-ETDRS quadrants (r = 0.790; P = 0.011) (Fig. 3). Additionally, 15/46 eyes (32.6%) had focal or pan-retinal laser treatment. Laser scars were visible on SD-OCT in 6 eyes, among which ERP was noted at the edges of laser scars in 5 cases.

#### Age-related macular degeneration

**Fig. 4:**
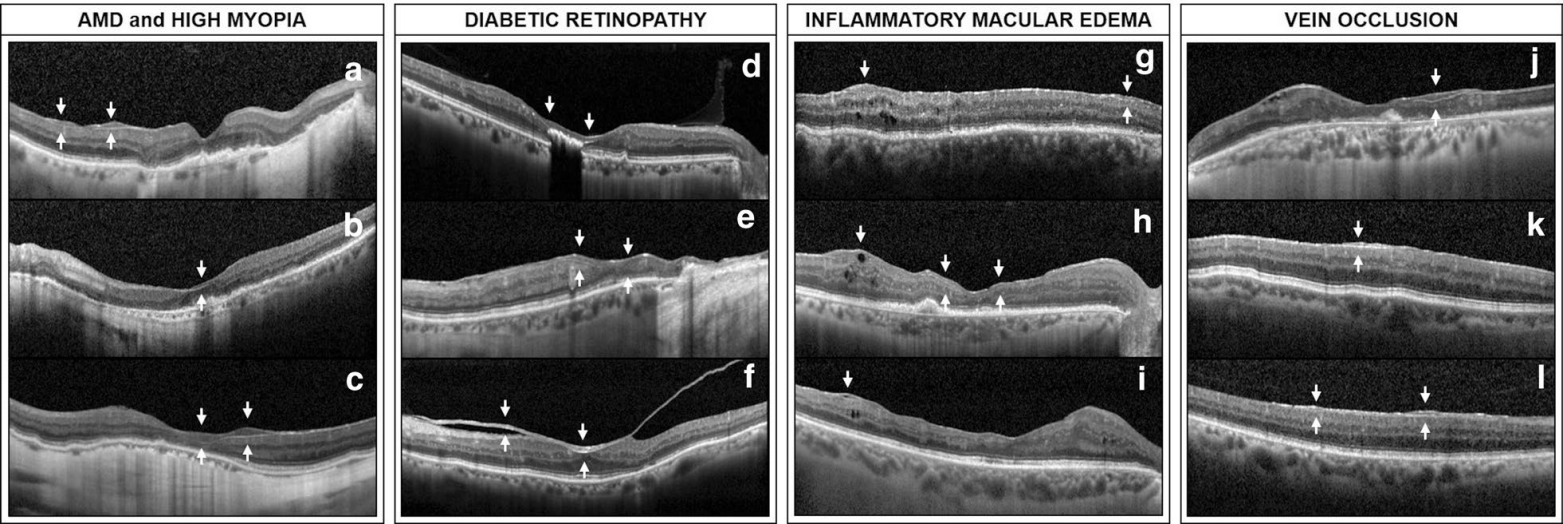
SD-OCT scans of eyes with retinal diseases and epiretinal proliferation. **a**, **b** Spectral-domain optical coherence tomography (SD-OCT) scans of patients with AMD showing epiretinal proliferation (ERP) over the retinal surface (white arrow). **c** Highly myopic eye with posterior staphyloma. On SD-OCT, ERP was noted in the central and inner temporal macular area. **d** SD-OCT scan of a patient with proliferative diabetic retinopathy and history of macular edema. Diffuse ERP was noted in the central foveal area. **e** Proliferative tissue was noted along the edges of extensive laser scars in a patient who underwent vitrectomy and laser therapy for proliferative diabetic retinopathy (PDR). **f** Patient with PDR and vitreomacular adhesion with a thick posterior hyaloid, on which ERP is seen. **g**–**i** SD-OCT scans of eyes treated for macular edema related to chronic hypotony (**g**), and for chronic macular edema post retinal detachment repair (**h**, **i**). ERP was noted over the retinal area showing focal chronic intraretinal cysts. Cystic cavities were noted to affect the proliferative tissue (**h**, **i**). **j** SD-OCT scan of a patient with central retinal vein occlusion (CRVO) showing ERP in area of retinal thinning and outer retinal layers disruption. **k**, **l** Patients with branch retinal vein occlusion (BRVO) showing ERP in the retinal area corresponding to the affected vein branch

Eight out of fourteen eyes (57.1%) with AMD were treated by anti-vascular endothelial growth factor (VEGF) injection for active neovascularization (Fig. 4a). On SD-OCT scans, 10 of 14 eyes (71.4%) displayed pigment epithelium detachment (PED). In eyes with PED, localization of ERP within the different ETDRS quadrants was correlated with localization of PED (r = 0.847; P = 0.004).

#### Diabetic Retinopathy

Ten eyes demonstrated diabetic retinopathy, of which 9 (90.0%) had proliferative diabetic retinopathy (PDR) (Figs. 4d–f), and one (10.0%) eye had severe non-proliferative diabetic retinopathy (NPDR). All diabetic eyes had a history of macular edema. Eight of 10 (80.0%) eyes received anti-VEGF intravitreal injection, 1/10 (10.0%) received intravitreal dexamethasone implant and 1/10 (10.0%) received both. Additionally, 9 out of 10 (90.0%) eyes had pan-retinal laser photocoagulation, and two eyes (20.0%) also had focal macular laser treatment.

#### Refractory chronic macular edema

Among the 10 eyes with refractory macular edema, 5 (50%) eyes had chronic macular edema after retinal detachment repair (Fig. 4h), 3 (30%) eyes had refractory pseudophakic macular edema (Fig. 4g), 1 (10%) eye was followed for idiopathic posterior uveitis, and 1 (10%) eye had hypotony maculopathy (Fig. 4i). All of these eyes presented chronic macular edema and had limited visual acuity [0.74 ± 0.69 (range: 0.1–1.98) LogMAR, (20/110 Snellen equivalent)]. Three of ten (30.0%) eyes had been treated with anti-VEGF therapy, 3/10 (30.0%) with intravitreal dexamethasone implant and 2/10 (20.0%) with both.

#### Vein occlusion

Two eyes had a central retinal vein occlusion (CRVO) (Fig. 4j), and six eyes had a branch retinal vein occlusion (BRVO) (Figs. 4k, l). In eyes with BRVO, epiretinal proliferation was systematically noted in the retinal area corresponding to the occluded branch vessel. All eyes were treated for macular edema, among which 5 received anti-VEGF injection, 1 received dexamethasone implant and 1 received both. Six out of 10 eyes (60%) had previous pan-retinal laser photocoagulation.

#### High myopia

Finally, four eyes had a high myopia with posterior staphyloma, but without other retinal conditions or history of choroidal neovascularization (Fig. 4b, c).

## Discussion

Epiretinal proliferation has been described in LMH and FTMH, where the alteration typically occurs at the edges of the hole [1, 7, 8]. More recently, Itoh and colleagues reported its presence in association with ERM development [3]. In their study, they reported hyporeflective proliferation in 19 out of 3291 eyes with ERM. Consistently, co-existence of both ERM and ERP has been demonstrated by Schuman and co-workers [9]. Using SD-OCT imaging and ultrastructural analysis, they noticed presence of both tissues with close proximity in a two-layer complex, in different vitreoretinal pathologies. In the present paper, we identified ERP in eyes with different VRI disorders, but also in eyes with various retinal conditions, including vascular, inflammatory and degenerative diseases, using SD-OCT. This suggests that different signals may trigger this proliferative process [10].

Histological studies in LMH have reported that ERP consists of retinal glial cells, specifically Müller cells, and lacks myofibroblasts [5, 9]. Conversely, idiopathic ERM consists mainly of hyalocytes, fibroblasts and myofibroblasts, which explain their tractional properties. Macroscopically, ERP had a yellow appearance when in the foveal area, which may be explained by the presence of xanthophyll pigments, as demonstrated by Obana and colleagues [9, 11]. This reinforces the suggestion that Müller cells are the main constituent of ERP, and that it originates from the retinal tissue [12]. Nevertheless, the exact pathogenesis of ERP remains poorly understood. One suggestion was that this proliferation develops in association with inner retinal defects [1]. Consistently, in eyes with LMH, FTMH or iatrogenic inner retinal defects, ERP was present on the retinal surface at the edges of the lesions. However, it remains unclear if the proliferation is initially originating from the retinal defect, or growing over the retinal surface toward the retinal defect. In all LMH eyes, ERP was found to be connected with retinal layers within the hole, rendering the latter hypothesis unlikely. In ERM eyes, as in eyes within the retinal diseases group in the present study, no break within the retinal tissue were visible, and therefore a clinically significant retinal defect does not seem to be a required condition for ERP development. However, focal opening in the internal limiting membrane induced by vitreous traction may be present, even if undetectable by SD-OCT examination [13]. Alternatively, it is possible that stretching of glial Müller cells by oblique anteroposterior vitreous traction in case of FTMH, or tangential traction in case of ERM may be sufficient mechanical factors to activate glial cells leading to ERP development [14, 15].

After macular peeling surgery, ERP was noted at the edges of inner retinal defect, but also in association with dissociation of optic nerve fiber layer (DONFL), visualized on OCT scans as retinal dimples. This change in inner retinal morphology is thought to be due to injury to the Müller cell footplates by peeling off the internal limiting membrane [16, 17]. While inner retinal dimples tend to increase over the first 6 months after surgery, it has been shown that their appearance decreases after 12 months, suggesting a gradual regeneration of Müller cell processes [18]. Thus, the mechanical stimuli induced by ILM peeling may trigger reactive gliosis, which may be sufficient to explain ERP development.

Epiretinal proliferation was also present in eyes with various retinal diseases, and without apparent VRI abnormalities. Interestingly, in eyes with macular edema, localization of ERP within the different ETDRS quadrants matched with the morphological distribution of intraretinal fluid. Müller cells are known to be one of the key player controlling retinal homeostasis and fluid movement into and out of the retina [19]. Chronic accumulation of intraretinal fluid may over-stimulate Müller cells, and may lead to expansion of retinal tissue and stretching of the Müller cells, contributing to glial cells hypertrophy and proliferation [15, 20]. While macular edema was associated with ERP development, 6/14 (42.9%) eyes with AMD had no history of macular edema. Hence, additional stimuli may have contributed to glial cells stimulation, and we notably found that the localization of ERP within the ETDRS quadrants was correlated with the localization of PED. Even though the role of retinal glial cells in AMD pathogenesis has been sparsely studied, astrocytes and Müller cells are known to play a role in AMD development, with notably gliosis of Müller cells [21, 22]. Almost a third of eyes in retinal diseases group had a history of retinal laser treatment, and we noted in five eyes presence of proliferative tissue along the border of the laser scars. Thus, retinal laser treatment seemed to induce a gliotic reaction. Consistent with this hypothesis, a previous experimental study noted activation of Müller cells at the burn site of laser, followed by proliferation and migration of these glial cells [23].

Overall, presence of ERP was found to be correlated with a variety of significant retinal alterations, including partial inner or full-thickness retinal defect, ERM, intraretinal cysts, PED and outer retinal layers disruption. The most frequently involved retinal quadrant was the 1-mm central subfield (as for the retinal pathology), and ERP was also more frequently present in the temporal than the nasal macula. This could be explained by a greater Müller cell densities in the central fovea and temporal retina [24]. However, ERP may also be found in paracentral macula away from the foveal center in cases of extrafoveal iatrogenic retinal defect. Hence, development of ERP and its location within the retina seemed more correlated with the presence of any retinal lesions [8]. Indeed, all retinal injuries may potentially trigger hypertrophy, proliferation and migration of Müller cells [20].

This Müller cell gliosis may either have protective or toxic effects on photoreceptors and retinal homeostasis [10, 20]. While proliferative gliosis may contribute to acceleration of neurodegeneration in chronic diseases such as AMD and diabetic retinopathy; activated Müller cells may also have neuroprotective effect after retinal injury. Indeed, there is emerging evidence suggesting that Müller glial cells are dormant stem-like cells with regenerative properties [25]. Therefore, development of ERP in eyes with different retinal conditions may represent a spontaneous cellular attempt to protect the retinal tissue from further damage, and to contribute to retinal tissue repair [25, 26].

Limitations of our study are its cross-sectional and non-comparative design, and the lack of scans in the outer macular area in a subset of patient. While a 30 × 25-degree macular raster was required to assess the presence of proliferation within the different ETDRS quadrants, some patients had only a 30 × 15-degree macular raster, and therefore the rate of superior and inferior outer sectors involved may have been underestimated. Absence of longitudinal evaluation did not permit evaluation of whether ERP development contributed to retinal repair or rather to progressive retinal degeneration, and the lack of comparative group did not allow evaluation of potential risk factors for ERP development. However, this descriptive study did include a broad spectrum of VRI and retinal conditions, in order to demonstrate that this proliferative tissue is not limited to LMH and FTMH, but may also occur in response to various retinal alterations. Further studies including a more homogenous population would be necessary to study specific risk factors and prognostic factors for ERP development.

In conclusion, this study highlights the presence of ERP in eyes with various conditions. While this proliferative tissue was initially described as a marker of severity for LMH/FTMH, it may actually result from a regenerative process involving Müller glial cells proliferation in response to retinal injury. Virtually all retinal pathogenic stimuli may activate Müller cells, and therefore, ERP may occur in response to different stimuli such as retinal edema, laser retinopexy, or choroidal neovascularization.

## Data Availability

All deidentified and coded data of patients included in the study are available by request.

## Abbreviations

AMD: Age-related macular degeneration
BRVO: Branch retinal vein occlusion
CRVO: Central retinal vein occlusion
DONFL: Dissociation of optic nerve fiber layer
ERP: Epiretinal proliferation
ERM: Epiretinal membrane
ELM: External limiting membrane
ETDRS: Early treatment diabetic retinopathy study
EZ: Ellipsoidal zone
FTMH: Full-thickness macular holes
LHEP: Lamellar hole-associated epiretinal proliferation
LMH: Lamellar macular hole
MacTel: Macular telangiectasia
LogMAR: Logarithm of the minimum angle of resolution
PED: Pigmentary epithelium detachment
PDR: Proliferative diabetic retinopathy
SD-OCT: Spectral-domain optical coherence tomography
SD: Standard deviation
VEGF: Vascular endothelial growth factor
VRI: Vitreoretinal interface
